# Identification of hospital patients in need of palliative care – a predictive score

**DOI:** 10.1186/s12904-016-0094-7

**Published:** 2016-02-22

**Authors:** Cornelia Meffert, Gerta Rücker, Isaak Hatami, Gerhild Becker

**Affiliations:** 1 Clinic for Palliative Care, Medical Center, University of Freiburg Faculty of Medicine, University of Freiburg, Robert-Koch-Str. 3, 79106 Freiburg, Germany; 2 Institute for Medical Biometry and Statistics, Medical Center - University of Freiburg, Stefan-Meier-Str. 26, 79104 Freiburg, Germany; 3 Department of Anesthesiology, Intensive Care and Pain Therapy, St. Vincent Hospital Landau, Cornichonstr. 4, 76829 Landau, Germany

**Keywords:** Palliative medicine, Terminally ill patients, Palliative care needs, Palliative care

## Abstract

**Background:**

Palliative care was initially developed for patients with advanced cancer. Over the past years, however, palliative care has broadened its focus from terminal cancer patients to patients with other serious, life-limiting illnesses. Nevertheless, the identification of palliative care needs (PCNs) among hospital patients remains an insufficiently investigated topic of research. The aim of our study was to describe the characteristics of hospital patients with palliative care needs and to develop a score for their identification.

**Methods:**

We conducted an epidemiological study. Data were collected prospectively from inpatients at the University Medical Center Freiburg, Germany. For each patient discharged from a hospital ward, the treating physician had to report whether the patient had PCNs or not. The response rate was 96 %, and data from 39,849 patients could be analyzed. A binary logistic regression analysis was performed in order to identify risk factors for developing PCNs and to develop a predictive score for the identification of patients with PCNs upon their admission to the hospital. In order to validate the risk prediction model, we used a bootstrap analysis.

**Results:**

During the study period, 6.9 % (2757) of all patients had palliative care needs. Only 56 of them (2 %) received palliative treatment. Binary logistic regression analysis showed that older patients without relatives who suffered from metastatic cancer and/or liver cirrhosis had the highest risk of developing palliative care needs (PCN-score; sensitivity: 0.815; specificity: 0.640).

**Conclusions:**

Given the aging population and associated increase in the number of patients requiring palliative care, it is crucial to detect palliative care needs in hospital patients with both cancerous and non-cancerous life-limiting diseases. Our predictive score contributes to the identification of palliative care needs in patients with life-limiting diseases, which allows physicians to take the appropriate therapeutic steps.

## Background

In industrialized countries, 75 % of deaths are caused by progressive advanced chronic diseases [1]. Furthermore, it is estimated that 63 % of those who die may need palliative care [2]. We are faced with an aging population that is expected to age even further in the future [3]. In 2009, 17 % of Europe’s total population was aged ≥ 65 years. Experts predict that this age group will account for 29 % of all European citizens by 2050 [4]. In light of these figures, we assume that the prevalence of advanced malignancies and chronic diseases will continually increase. Therefore, one can also expect a growing number of patients in need of end-of-life care [5].

The medical focus of palliative care is symptom-related treatment aimed at reducing pain, physically burdensome symptoms (e.g., dyspnea, nausea), and psychological afflictions (e.g., depression). Rather than prolonging life, treatment aims at enhancing quality of life (QoL). Palliative care is a comprehensive therapeutic approach that encompasses the treatment of physical symptoms as well as the integration of psychological, social, and spiritual needs of both patients and their relatives. Ideally, palliative care should be implemented as soon as an incurable disease is diagnosed, not only in the imminent terminal phase [6].

Palliative care was initially developed for patients with advanced cancer and an increasing number of cancer patients receive palliative care alongside standard oncology care. However, it cannot be denied that other patients are also in need of palliative care. Over the past years, palliative care has broadened its focus from terminal cancer patients to patients with other serious, life-limiting illnesses [7], such as terminal cardiac, hepatic, or respiratory insufficiency, human immunodeficiency virus (HIV), or amyotrophic lateral sclerosis (ALS).

Nevertheless, the identification of palliative care needs (PCNs) in hospital patients remains an insufficiently investigated topic of research. Therefore, the main goals of our study were to describe the characteristics of hospital patients with PCNs, to identify risk factors for the development of palliative care needs and to develop a score for their identification.

## Methods

### Data Sampling

Data were collected prospectively from patients at the University Medical Center Freiburg (UMCF), Germany, one of the largest hospitals in Europe, with a total of 1479 hospital beds in 42 clinical departments. Sampling took place from January 2004 to May 2005. At the time of the study, the hospital had no specialized palliative care service for in-house consultations and no palliative care unit.

For each patient discharged or transferred from a UMCF ward or deceased, electronic data sampling by the attending physician was mandatory. For the purposes of our study, we modified the file mask of the electronic discharge management by adding the dichotomous question: “Does or did this patient have palliative care needs?” For each patient, this question had to be answered by the treating physician responsible for discharge management. When the physician clicked on the question, a text box appeared explaining how PCN was defined using the WHO definition: “Palliative care is the active total care of patients whose disease is not responsive to curative treatment. Control of pain, of other symptoms, and of psychological, social, and spiritual problems is paramount.” [8] Some aspects of palliative care needs (e.g., pain, fatigue, dyspnoea, nausea/vomiting, constipation, anxiety, and depression) were also specified in the text box. Without answering the question about PCN, the electronic discharge management could not be completed, thereby guaranteeing a nearly 100 % response rate.

During the study period, data were collected from 100,679 records. Data collection also included patients who were transferred to another ward or another department within the hospital. To prevent a bias toward patients who had longer hospital stays and were transferred to different wards several times, only the data from the last ward before leaving the hospital were included. This limited data to 39,849 cases. If a patient had two or more hospital stays during the observation period, we analyzed each hospital stay separately. As a result, a total of 39,849 hospital stays were analyzed for 26,767 different patients treated in the clinic. A total of 13,082 (32.8 % of 39,849) cases resulted from readmissions.

Patients who died during their hospital stay were included in our data.

Details of the study design are described elsewhere [5].

### Study questions and statistical analyses

The main outcome measures of the study were the identification of the percentage of patients with PCNs in a large acute care hospital and the development of a score for their identification.

Data were analyzed with SPSS software (IBM SPSS for Windows, Version 21.0). The *t*-test was used for interval scaled variables; associations between dichotomous variables were tested with the χ^2^ statistic. In addition, a binary logistic regression analysis was performed in order to identify risk factors for developing PCNs and to develop a predictive score for the identification of patients with PCNs upon their admission to the hospital. The resulting score was interpreted as the estimated probability that a patient develops PCNs. Patients’ PCN status could be divided into two groups (yes or no). Based on variation of the cutoff, pairs of false positive rate (1-specificity) and true positive rate (sensitivity) were presented in a receiver operating characteristic (ROC) curve. In order to validate the risk prediction model, we used a bootstrap analysis, i.e., repeated sampling with replacement and modeling [9].

All tests were two-tailed, and *p* < .05 was considered statistically significant.

### Ethics

The study protocol was approved by the Ethics Committee of the University Medical Center Freiburg and the data security official. Patients gave written consent to use their routinely collected data for scientific purposes. Regulations of the European Data Protection Directive [10] were followed. The study was conducted according to the Declaration of Helsinki [11].

## Results

### Patients with PCNs and their characteristics

During the study period, 6.9 % of all patients (2757/39,849) had PCNs. Patient characteristics such as the presence of PCNs, number of days in the hospital, and palliative treatment as a part of their therapy are listed in Table 1.

**Table 1:** Patient characteristics, palliative treatment, hospital stay, and discharge status

Characteristic	Patients with PCNs	Patients without PCNs	*p*
Patients, N (%)	2757 (6.9)	37,092 (93.1)	
Age in years, mean ± SD	63.8 ± 14.5	56.5 ± 18.1	<.001
Male patients, N (%)	1569 (56.9)	20,207 (54.5)	.013
Presence of relatives, N (%)	1596 (57.9)	22,287 (60.1)	.023
Palliative treatment, N (%)	56 (2.0)	60 (0.2)	<.001
Hospital stay in days, mean ± SD	12.1 ± 14.6	8.6 ± 10.7	<.001
Deceased, N (%)	125 (4.5)	940 (2.5)	<.001
Discharged home, N (%)	2086 (75.7)	30,472 (82.2)	<.001
Discharged to another acute care hospital or rehabilitation center, N (%)	460 (16.7)	4844 (13.1)	<.001
Discharged elsewhere (e.g. nursing home), N (%)	86 (3.1)	836 (2.3)	.004

Abbreviations: *PCN* palliative care need, *SD* standard deviation

Only 56 out of 2757 patients (2 %) with PCNs received palliative treatment in the sense of non-curative, disease-modifying therapies such as palliative radiation therapy or the palliation of breathlessness in advanced COPD. Patients with PCNs were significantly older than those without (*p* < .001) and stayed on average 3.5 days longer in the hospital. Significantly more patients with PCNs died during their hospital stay than those without (4.5 % v 2.5 %; *p* < .001) (Table 1). The proportion of patients with and without PCNs and the frequency of PCNs in each diagnostic group are shown in Table 2.

**Table 2:** Proportion of patients with and without PCNs and frequency of PCNs in each diagnostic group

	Patients with PCNs (*N* = 2,757)	Patients without PCNs (*N* = 37,092)	*p*	Proportion of patients with PCNs relative to all patients of diagnostic group (%)
	*N* (%)	*N* (%)		
Metastases	1068 (38.7)	3188 (8.6)	<.001	25.1
Cancer	1836 (66.6)	9748 (26.3)	<.001	15.8
Anemia	373 (13.5)	2399 (6.5)	<.001	13.5
Acute renal failure	99 (3.6)	670 (1.8)	<.001	12.9
Liver cirrhosis	122 (4.4)	868 (2.3)	<.001	12.3
Dementia	58 (2.1)	416 (1.1)	<.001	12.2
Depression	107 (3.9)	999 (2.7)	<.001	9.7
Chronic renal failure	283 (10.3)	2667 (7.2)	<.001	9.6
AIDS/HIV	11 (0.4)	103 (0.3)	.250	9.6
Chronic heart failure	178 (6.5)	1692 (4.6)	<.001	9.5
Diabetes mellitus	302 (11.0)	3537 (9.5)	.015	7.9
COPD	132 (4.8)	1323 (3.6)	.001	9.1
Cardiomyopathy	59 (2.1)	691 (1.9)	.302	7.9
Coronary heart disease	436 (15.8)	5840 (15.7)	.923	6.9
Apoplectic insult	74 (2.7)	1613 (4.3)	<.001	4.4

Note that the percentages in the first two columns do not equal 100 %, as diagnostic groups are not disjunctive

Abbreviations: *PCN* palliative care need

According to our data, the majority of patients with PCNs had cancer (66.6 %, 1,836/2757). The prevalence of PCNs was highest in patients with metastases (25.1 %, 1068/4256), malignant neoplasm (15.8 %, 1836/11,584), anemia (13.5 %, 373/2772), acute renal failure (12.9 %, 99/769), liver cirrhosis (12.3 %, 122/990), and/or dementia (12.2 %, 58/474). In contrast, patients with cardiomyopathy (7.9 %, 59/750), coronary heart disease (6.9 %, 436/6276), or apoplectic insult (i.e. intracerebral hemorrhage, cerebral infarction, stroke, or sequelae of cerebrovascular disease) (4.4 %, 74/1687) had a lower rate of PCNs.

### Binary logistic regression analyses

In order to identify risk factors for developing PCNs and to develop a score for the identification of patients with PCNs, we conducted a binary logistic regression analysis. In the regression model, we included sex, age, incurable illnesses ranking among the top reasons for mortality [12], and sociological factors such as the presence/absence of relatives.

When a patient is admitted to the hospital, information on all variables included (as mentioned above) should be available. Therefore, data concerning therapy and discharge were not included. Since the patient’s level of care at admission was only registered in 23,851 cases, a high level of care (summary of level of care at admission ≥ 5) was not taken into consideration here.

The regression analysis (based on the initial set of variables) showed that a diagnosis of cancer was the highest risk factor for developing PCNs, with an odds ratio of 3.45. Furthermore, scoring positive for metastases contributed to an increased risk by a factor of 3.29. The third greatest risk factor was dementia, followed by HIV and liver cirrhosis. Based on the entire sample with 39,849 cases, Table 3 shows the initial model with all variables included in the regression analysis.

**Table 3:** Risk factors for developing palliative care needs (PCNs)

Risk factor	N	Odds ratio (95 % CI)	*p*
Cancer	11,584	3.45 (3.10 – 3.84)	<.001
Metastases	4256	3.29 (2.96 – 3.65)	<.001
AIDS/HIV	114	3.23 (1.69 – 6.16)	<.001
Dementia	474	2.12 (1.58 – 2.85)	<.001
Liver cirrhosis	990	2.06 (1.67 – 2.53)	<.001
Acute renal failure	769	1.88 (1.50 – 2.36)	<.001
Absence of relatives	15,966	1.52 (1.39 – 1.65)	<.001
Chronic heart failure	1870	1.52 (1.27 – 1.81)	<.001
Anemia	2772	1.49 (1.32 – 1.69)	<.001
Depression	1106	1.49 (1.20 – 1.85)	<.001
Cardiomyopathy	750	1.41 (1.05 – 1.88)	.021
Chronic renal failure	2950	1.26 (1.09 – 1.45)	.001
Coronary heart disease	6276	1.14 (1.02 – 1.29)	.028
Sex: female	18,073	1.03 (0.95 – 1.13)	.442
Age [1/y]	39,849	1.02 (1.02 – 1.02)	<.001
Diabetes mellitus	3839	0.97 (0.85 – 1.11)	.674
COPD	1455	0.97 (0.80 – 1.18)	.788
Apoplectic insult	1687	0.84 (0.65 – 1.07)	.151

Data base: *N* = 39,849

A second analysis of the data, including the level of care (23,851 cases), showed that a high level of care at admission was a highly significant risk factor for PCNs, which was to be expected. With regard to significance, a high level of care ranked third in the list of potential risk factors (odds ratio 2.17; 95 % CI, 1.92 to 2.45; *p* < .001).

In order to develop a manageable and easy to compute predictive score for the identification of PCNs in hospital patients, we calculated the final model (based on these 23,851 cases), only taking into account the six most important risk factors that were included in the binary logistic regression analysis with forward selection by step 1 to step 6 (cancer, metastases, age, absence of relatives, liver cirrhosis, and high level of care at admission). After step 6 the improvements in the explained variance (Nagelkerkes R^2^) were minimal. Because we were interested in having a sparse model to avoid overfitting, we restricted our score to these first six variables. Table 4 shows the equation of the model with the regression coefficients and standard errors.

**Table 4:** Predictive score – equation of the model

Variables	Regression coefficient B	Standard error	Wald	*p*	Exp(B)	95 % confidence interval exp(B)	
						lowest	highest
Cancer (*x1*)	1.502	.074	406.934	.000	4.490	3.880	5.196
Metastases (*x2*)	1.054	.079	178.327	.000	2.870	2.458	3.350
Age [1/y] (*x3*)	0.026	.002	154.769	.000	1.026	1.022	1.030
Absence of relatives (*x4*)	0.421	.062	46.160	.000	1.523	1.349	1.720
Liver cirrhosis (*x5*)	0.895	.132	46.176	.000	2.448	1.891	3.169
High level of care (*x6*)	0.809	.062	172.559	.000	2.245	1.990	2.533
Constant	−5.762	.147	1542.663	.000	.003		
Equation (Nagelkerkes R^2^: 0.199)	$$ \begin{array}{c}\hfill \mathrm{p} = 1\ /\ \left(1+{\mathrm{e}}^{-\mathrm{z}}\right)\hfill \\ {}\hfill \mathrm{z} = -5.762 + 1.502\ *x1 + 1.054\ *x2 + 0.026\ *x3 + 0.421\ *x4 + 0.895\ *x5 + 0.809\ *x6\hfill \end{array} $$						

**Fig. 1:**
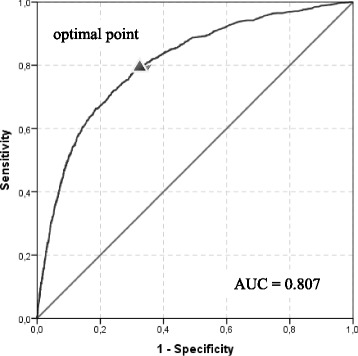
Receiver operating characteristic (ROC) curve

The distribution of parameters after conducting 1000 bootstrap replications showed a close agreement between the original data set and the bootstrap results (data not shown). Figure 1 illustrates the results of the regression analysis using an ROC curve.

The area under the curve (AUC) represents the probability that the assay result for a randomly chosen positive case will exceed the result for a randomly chosen negative case. The AUC was calculated at 0.807 (*p* < .001; 95 % CI, 0.795 to 0.819). To ensure a good sensitivity, we chose 0.04 as the cutoff point, which led to a sensitivity of 0.815 and a specificity of 0.640. Thus, approximately 81.5 % of all patients with PCNs would be correctly identified as such, and 36.0 % of all patients without PCNs would be incorrectly identified as having PCNs.

Due to the low prevalence of 6.9 %, the positive predictive value was low (14.4 %), while the negative predictive value was high (97.9 %), which emphasizes the envisaged role of the score as a "rule out" test for PCN (predictive values calculated via Bayes' theorem).

Our results showed that older patients without relatives who have a high level of care at admission and suffer from metastatic cancer and/or liver cirrhosis had the highest risk of developing PCNs.

## Discussion

According to the World Health Organization (WHO), palliative care represents “an approach that improves the quality of life of patients and their families facing the problems associated with life-threatening illness, through the prevention and relief of suffering by means of early identification and impeccable assessment and treatment of pain and other problems, physical, psychosocial and spiritual” [6]. Thus, palliative care primarily aims at improving QoL of both patients with incurable diseases and their relatives. Erroneously, palliative care is often reduced to end-of-life care [13, 14]. It is also predominantly offered only after life-prolonging treatment has failed [15]. However, a substantial number of patients with incurable diseases suffer from a number of physically distressing symptoms (i.e. pain) as well as psychosocial and spiritual burden. This suffering may be overlooked if only life-prolonging therapy is offered [16]. There is substantial evidence that the integration of palliative care – either in combination with standard care or as the main focus of care – leads to better patient and caregiver outcomes and a reduced use of futile intensive care [13]. Therefore, a shift from the continued use of ineffective therapies to a focus on palliative care and the relief of symptoms throughout the course of illness is widely encouraged [16].

For patients with non-small cell lung cancer (NSCLC), Temel et al. [17] showed that early palliative care (EPC) improved patients’ QoL, reduced the incidence of depression, and decreased the number of aggressive (and generally futile) therapies at the end of life. In addition, EPC increased patients’ survival time [17]. In a randomized study with patients suffering from different cancer entities, Zimmermann et al. [18] found that patients who received EPC not only had a better QoL but also a higher level of satisfaction with their treatment compared to those in the control group, who were not offered EPC.

One of the main goals of our study was to identify the percentage of patients with PCNs in a large acute care hospital and to describe their characteristics. Our results showed that predominantly but not exclusively patients with metastatic cancer had a high risk of developing PCNs. However, patients with other non-cancerous illnesses were also in need of palliative care. For over 15 years, experts have been promoting the idea that palliative care should be provided on the basis of need rather than diagnosis [19]. In light of such demands, the findings of our study confront us with rather alarming figures; only 2 % of patients with PCNs actually received palliative treatment, in 98 % these needs remained unmet.

Another focus of our study was the development of a predictive score to aid in the identification of PCNs. We hope that the score aims at increasing physicians’ awareness of palliative care needs – without being asked explicitly: “Does this patient have a palliative care need?” Our results showed that older patients without relatives who had a high level of care at admission and suffered from metastatic cancer and/or liver cirrhosis had the highest risk of developing PCNs. With a sensitivity of 0.815 and a specificity of 0.640, this score is not optimal and it remains unclear how to deal with patients whose PCNs either are not identified or are falsely identified. One simple and obvious solution to this problem is to ask patients directly whether or not they perceive a need for specialized (palliative) care (i.e., treatment of pain and other problems, physical, psychosocial and spiritual [6]) in addition to standard care.

Overall, our regression models confirmed the general hypothesis that PCNs are largely found in patients with cancerous diseases, particularly in the metastatic stage [20]. However, patients who suffer from severe chronic diseases (e.g. liver cirrhosis, AIDS-HIV or renal failure) and have a very poor general condition and a high need of care are also at risk of developing PCNs. Furthermore, the absence of relatives and the presence of psychological ailments like dementia and depression were shown to be clear risk factors, which underlines the importance of psychosocial issues in palliative care.

### Limitations

Our study had a number of inherent limitations. Our data are about 10 years old, and at the time of the study, the hospital had no specialized palliative care service for in-house consultations and no palliative care unit. Nowadays, an increasing number of patients –above all cancer patients – receive palliative care alongside standard care. Today, physicians’ awareness of patients’ PCNs is higher than 10 years ago, and if asked again, they would probably identify more patients with PCNs.

Furthermore, we asked the treating physician about patients’ PCNs and not the patient himself or his/her relatives. As we conducted an epidemiologic study with close to 40,000 patients, the latter was not possible. This might be a source of bias and a second reason why the rate of PCNs might have been underestimated.

A current study quantified the number of hospital patients who are within the last year of life by connecting data from the hospital system with national death registration data [21]. The authors identified about a third of hospital inpatients who have entered the last year of their lives. However, [21] was a prevalent cohort study and thus prone to length-biased sampling, meaning that patients with longer hospital stays were more likely to be sampled. Using this design, mortality is likely to be overestimated, as the length of hospital stay is associated with disease severity, mortality and PCNs.

A definition of PCNs is complex because palliative care can be divided into two levels. First, there is general palliative care that can be provided by generalists in their daily work. Second, there are specialist palliative care physicians and nurses who are trained specifically to handle complex palliative situations. To keep the survey clear and simple, we intentionally used the widely accepted WHO definition of palliative care [22] without differentiating between a primary and a secondary level of palliative care. To what extent generalists may satisfy basic needs for palliative care and how much specialist palliative care is needed for complex situations cannot be answered by our data.

In the database, patients with multiple stays in the hospital were analyzed as separate cases. This may have been a source of bias. Conversely, though, analyzing multiple stays of the same person as only one case could also present a source of bias. Which stay should be regarded as the relevant one? In order to more accurately calculate the number of beds needed for patients with PCNs, we decided to analyze multiple stays as independent.

With regard to our predictive score, we must acknowledge certain limitations. The risk prediction model was developed using the same data set that it was applied to afterwards. It is well known that this leads to overfitting, meaning that the explained variance may be overestimated and the appraised predictive ability of the model may be too high. To investigate the predictive ability of our score, we conducted a bootstrap analysis. With respect to the estimated standard errors, we found close agreement between the original data set and the bootstrap results. Thus, it appears unnecessary to doubt the risk prediction model obtained in the original analysis.

We chose 0.04 as our cutoff point, meaning that a patient is deemed to be in need of palliative care if his/her probability score exceeds 0.04. This seemingly low threshold was due to the low prevalence of 6.9 % and the desire for high sensitivity at a low cost to specificity. This, however, may procure overestimated sensitivity and specificity, and conceivably our predictive score may be less perfect for future patients.

Prediction models in medicine have gained substantially more importance in recent years, and health care providers and policy makers are increasingly recommending the use of prediction models [23]. Above all, our predictive score aims at increasing the awareness of PCNs in patients with cancerous and non-cancerous life-threatening diseases. To what extent this score can be applied in the clinical setting and how it actually influences clinicians’ decision making and behavior as well as patient outcomes should be evaluated in further studies.

## Conclusion

Given the aging population and associated increase in the number of patients requiring palliative care [24], it is crucial that PCNs are detected in patients with cancerous and non-cancerous life-threatening diseases. In general, such patients are likely to suffer from pain, dyspnea, and other burdensome physical symptoms and require psychosocial or spiritual support in the course of their progressing disease [25]. These patients are in need of palliative care not only in the final months of their life but throughout the course of their disease.

Our predictive score contributes to the identification of PCNs in patients with life-limiting diseases, which allows physicians to take appropriate therapeutic steps. This not only involves pain and symptom management but also attention to psychosocial and spiritual needs, such as being treated as a “whole person”, preparation for death, and achieving a sense of completion.

## Abbreviations

ALS: Amyotrophic lateral sclerosis
AUC: Area under the curve
DFG: German Research Foundation
EPC: Early palliative care
HIV: Human immunodeficiency virus
NSCLC: Non-small cell lung cancer
PCNs: Palliative care needs
QoL: Quality of life
ROC curve: Receiver operating characteristic curve
UMCF: University Medical Center Freiburg
WHO: World Health Organization
